# Global flyway evolution in red knots *Calidris canutus* and genetic evidence for a Nearctic refugium

**DOI:** 10.1111/mec.16379

**Published:** 2022-02-15

**Authors:** Jesse R. Conklin, Yvonne I. Verkuil, Phil F. Battley, Chris J. Hassell, Job ten Horn, James A. Johnson, Pavel S. Tomkovich, Allan J. Baker, Theunis Piersma, Michaël C. Fontaine

**Affiliations:** ^1^ Groningen Institute for Evolutionary Life Sciences (GELIFES) University of Groningen Groningen The Netherlands; ^2^ Wildlife and Ecology Group School of Natural Sciences Massey University Palmerston North New Zealand; ^3^ Global Flyway Network Broome Western Australia Australia; ^4^ Department of Coastal Systems NIOZ Royal Netherlands Institute for Sea Research Texel The Netherlands; ^5^ U.S. Fish & Wildlife Service, Migratory Bird Management Anchorage Alaska USA; ^6^ Zoological Museum Moscow MV Lomonosov State University Moscow Russia; ^7^ Department of Natural History Royal Ontario Museum Toronto Ontario Canada; ^8^ MIVEGEC, CNRS, IRD University of Montpellier Montpellier France; ^9^ Montpellier Ecology and Evolution of Diseases Network (MEEDiN) Montpellier France

**Keywords:** Bird migration, Climate change, Genetic differentiation, Genotyping‐by‐sequencing, Glacial refugia, Phylogeography, Population genomics

## Abstract

Present‐day ecology and population structure are the legacies of past climate and habitat perturbations, and this is particularly true for species that are widely distributed at high latitudes. The red knot, *Calidris canutus*, is an arctic‐breeding, long‐distance migratory shorebird with six recognized subspecies defined by differences in morphology, migration behavior, and annual cycle phenology, in a global distribution thought to have arisen just since the last glacial maximum (LGM). We used nextRAD sequencing of 10,881 single‐nucleotide polymorphisms (SNPs) to assess the neutral genetic structure and phylogeographic history of 172 red knots representing all known global breeding populations. Using population genetics approaches, including model‐based scenario‐testing in an approximate Bayesian computation (ABC) framework, we infer that red knots derive from two main lineages that diverged ca. 34,000 years ago, and thus most probably persisted at the LGM in both Palearctic and Nearctic refugia, followed by at least two instances of secondary contact and admixture. Within two Beringian subspecies (*C*. *c*. *roselaari* and *rogersi*), we detected previously unknown genetic structure among sub‐populations sharing a migratory flyway, reflecting additional complexity in the phylogeographic history of the region. Conversely, we found very weak genetic differentiation between two Nearctic populations (*rufa* and *islandica*) with clearly divergent migratory phenotypes and little or no apparent contact throughout the annual cycle. Together, these results suggest that relative gene flow among migratory populations reflects a complex interplay of historical, geographical, and ecological factors.

## 1 INTRODUCTION

The ecology and demography of species are typically viewed through the lens of present‐day or very recent (i.e., decades) observations and processes. However, it is clear that habitats, distributions, genetic diversity, and populations themselves are the legacies of events and conditions in historical or evolutionary time‐scales, and cannot be fully understood without a phylogeographic perspective (Avise et al., 1987; Knowles, 2009). This is particularly true for high‐latitude species, whose habitats were repeatedly transformed by Pleistocene glacial cycles, and whose current distributions may have arisen just since the last glacial maximum (LGM, c. 20,000 years before present, ybp; Hewitt, 2000). Of course, the effects of glaciations on historical distributions, and on population genetic diversity and structure, are strongly dependent on a species’ ecological and physiological attributes (Stewart et al., 2010).

Migratory birds are characterized by seasonal movements and high dispersal capabilities, with important consequences for population structure, the strength of geographical barriers, and the propensity to colonize novel ranges (Winker, 2010). The effects of glacial perturbations on the geographic organization of migratory bird species and subspecies are profound, although the precise consequences are varied and debated (Avise & Walker, 1998; Johnson & Cicero, 2004; Klicka & Zink, 1997; Weir & Schluter, 2004). For example, it remains unclear whether tundra‐specialized populations were most restricted and isolated (i.e., in “refugia”) during the LGM, when vast areas of the Arctic were under ice, or during the mid‐Holocene Climatic Optimum (c. 8,000 ybp), when a period of elevated temperatures limited the extent of tundra habitat (Arcones et al., 2020; Stewart & Dalén, 2008; Wauchope et al., 2017). Regardless, the present‐day global distributions of many arctic‐breeding migratory bird species attest to broad post‐glacial expansions (Kraaijeveld & Nieboer, 2000) and rapid colonization of new “flyways” (i.e., networks of routes and sites used throughout the annual journey) in response to changing conditions (Piersma, 2011).

**FIGURE 1:**
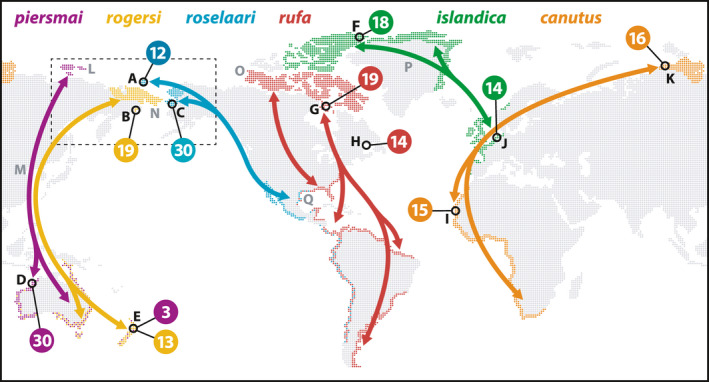
Global distribution and sampling of red knots. For each of six recognized subspecies (indicated by colour), arrows indicate general migration routes between breeding and (boreal) wintering areas (coloured areas). Numbers indicate total individuals sampled in each area (black circles). Letters refer to sampling sites (in black; Table S1 for details) and other locations mentioned in the text (in grey): A, Wrangel Island; B, SE Chukotka; C, Seward Peninsula; D, Roebuck Bay; E, Foxton Beach; F, Ellesmere Island; G, Southampton Island; H, Mingan Archipelago; I, Banc d’Arguin; J, Wadden Sea; K, Taimyr Peninsula; L, New Siberian Islands; M, Yellow Sea; N, Bering Sea (Land Bridge); O, Banks Island; P, Greenland Ice Sheet; Q, Gulf of Mexico. Dashed box indicates approximate extent of the Beringia region

Understanding the intertwining effects of historical and present‐day processes on migratory systems is best approached through population genetic and phylogeographic approaches based on genome‐wide markers, and informed by detailed ecological knowledge (Orsini et al., 2013). Among the most ecologically well‐studied migratory birds is the red knot, *Calidris canutus*, a globally‐distributed shorebird that breeds on high‐latitude (62–80ºN) arctic tundra and uses temperate and tropical intertidal mudflats during the rest of the year (Piersma, 2007; Piersma & Davidson, 1992). There are six recognized subspecies of red knot (Figure 1), distinguished by their breeding and nonbreeding ranges, and their migratory behavior, with one‐way distances ranging from <3,000 to >14,000 km (Piersma, 2007; Piersma, Rogers, et al., 2005) and including some of the longest non‐stop flights recorded in birds (Conklin et al., 2017). These populations differ in body size, plumage, and degree of spatial and behavioural overlap with neighbouring populations throughout the annual cycle (Buehler & Piersma, 2008; Tomkovich, 1992, 2001). Census population sizes vary by more than an order of magnitude (<20,000 to >400,000 individuals; Wetlands International, 2021), but most have faced significant declines in recent decades (Baker et al., 2004; Boyd & Piersma, 2001; Studds et al., 2017; van Gils et al., 2016), largely due to anthropogenic impacts on nonbreeding habitats (Baker et al., 2004; Piersma et al., 2016; Rakhimberdiev et al., 2015). Therefore, understanding the shared histories and magnitude of genetic, reproductive, and ecological separation among these populations is of significant conservation and evolutionary interest.

Observing shallow genetic differentiation and signals of historical bottlenecks in the mitochondrial control region (mtDNA), Buehler and Baker (2005) inferred a very recent origin of present‐day red knot populations, and a global expansion from a single LGM refugium (Buehler et al., 2006). Notably, some subspecies pairs that were indistinguishable in mtDNA (Buehler & Baker, 2005) have phenotypic differences in morphology and migration distance, timing, and direction. In red knots, migratory behaviour is further associated with seemingly “hard‐wired” endogenous annual rhythms in molt and mass, apparently adapted to flyway‐specific conditions (Karagicheva et al., 2016; Piersma, 2011). This suggests that complex, multitrait migratory “syndromes” can arise in ecological timescales, and in the presence of gene flow (Delmore et al., 2020; Pérez‐Tris et al., 2004). However, unravelling such recent, and potentially reticulated, evolutionary histories requires genome‐wide information (Brito & Edwards, 2009; Narum et al., 2014) coupled with powerful model‐based scenario‐testing such as approximate Bayesian computation (ABC) (Beaumont, 2010; Bertorelle et al., 2010; Hickerson et al., 2010).

Here, we revisit population structure and phylogeography of red knots, to describe the history of divergences and degree of neutral genetic differentiation among global flyway populations. For this, we exploit recent research on red knot ecology and migration, which has clarified spatiotemporal overlap among populations (e.g. Atkinson et al., 2005; Carmona et al., 2013; Nebel et al., 2000; Verhoeven et al., 2016) and has made available more comprehensive global sampling associated with known migratory phenotypes. We used nextera‐tagmented, reductively amplified DNA (nextRAD) sequencing (Russello et al., 2015) for *de novo* discovery of genome‐wide single‐nucleotide polymorphisms (SNPs) for population genetic analyses, and compared hypothesized evolutionary scenarios in an ABC framework using DIYABC (Cornuet et al., 2014). By reconstructing the recent evolutionary history of red knots, we (1) revise our understanding of LGM refugia and the colonization of global flyways, (2) reveal previously unrecognized population structure, and (3) provide a foundation for understanding how geography and ecology interact to regulate gene flow among migratory populations.

## 2 MATERIALS AND METHODS

### 2.1 Sampling and DNA extraction

We assembled DNA samples representing all recognized and hypothesized breeding populations within the global range of red knots (Figure 1, Table S1). Where possible, we used samples collected in known breeding areas; for *C*. *c*. *roselaari* (hereafter, we refer to populations by only their subspecific epithets) these included samples from two disjunct breeding areas: Wrangel Island, Russia and Seward Peninsula, Alaska, USA. We treated these as separate groups (*roselaari* West (W) and East (E), respectively), to test whether *roselaari* should be considered one or two independent demographic units. Because red knots breed in low densities in remote areas of arctic tundra, for some populations there were few or no breeding samples available. In these cases, we used samples collected from nonbreeding areas (i.e., sites used during migration and/or the boreal winter) when sampled individuals could be confidently assigned to breeding areas either because they were remotely tracked to breeding areas using light‐level geolocation, or because long‐term research programmes had established strong links between breeding and nonbreeding areas (e.g., through mark‐recapture/resight programmes). All tissue samples were acquired from museum collections or collected by the authors and colleagues in the field under all requisite permits appropriate to their respective countries and institutions.

We extracted genomic DNA from samples using three methods. For blood or organ tissue samples preserved in 95% ethanol, we used the DNeasy Blood and Tissue Kit (Qiagen) following the manufacturerʼs instructions for tissue. For blood samples preserved in Queenʼs lysis buffer, we used the NucleoSpin Blood QuickPure Kit (Macherey‐Nagel). For feather samples and blood stored on filter paper, we used ammonium acetate precipitation (Richardson et al., 2001). Extract quality was first assessed on a 1.5% agarose gel to exclude extractions with insufficient yield or excessively degraded DNA. We then quantified DNA concentrations using a Qubit 3.0 fluorometer (Life Technologies), diluted extracts to achieve relatively even concentrations, and dried down samples in a SpeedVac concentrator. We delivered 57–168 ng of DNA of 203 individual knots (Table S1) for SNP discovery and genotyping.

### 2.2 SNP genotyping using nextRAD sequencing

Genomic DNA was converted into nextRAD genomic fragment libraries (SNPsaurus, LLC, USA) following the method described by Russello et al. (2015). For each sample, 20–30 ng of genomic DNA was first fragmented with Nextera reagent (Illumina, Inc., USA), which also ligates short adapter sequences to the ends of the fragments. Fragmented DNA was then amplified, with primers matching the adapter and one primer extending 10 nucleotides into the genomic DNA with the selective sequence “GTGTAGAGCC”. Thus, only fragments starting with a sequence that can be hybridized by the selective sequence of the primer were efficiently amplified. PCR amplification was done at 74°C for 27 cycles. The nextRAD libraries were sequenced on an Illumina HiSeq‐4000 at the Genomics Core Facility, University of Oregon, USA.

Genotyping was performed using custom scripts (SNPsaurus, LLC). First, reads were trimmed in bbduk (BBMap tools; Bushnell, 2016). Next, a *de novo* reference was created from abundant reads (after removal of low‐quality (phred‐scale quality <20) and very high‐abundance reads) and reads that aligned to these. All 161,810,193 reads were mapped to the reference with an alignment identity threshold of 95% using bbmap (BBMap tools). Genotype calling was performed using SAMtools and BCFtools (samtools mpileup ‐gu ‐Q 10 ‐t DP, DPR ‐f ref.fasta ‐b samples.txt bcftools call ‐cv ‐ > genotypes.vcf), applying a minimum read depth filter of 7×. The genotype table was then filtered using VCFtools v.01.14 (Danecek et al., 2011) to remove SNPs called in <80% of samples and putative minor alleles with frequency <3% (allele counts <10), to exclude artefactual variants. The resulting VCF file included 4,911 unique loci (150‐bp sequences) containing 14,903 SNPs. Additional filtering was performed using VCFtools to remove indels (*n* = 452) and samples that failed to sequence (*n* = 13 individuals). Next, to exclude potential genotyping errors, SNPs deviating from Hardy‐Weinberg proportions in at least six of seven hypothesized populations were identified and removed using VCFtools *(p *< .05; *n* = 58 SNPs). To minimize linkage among loci, we used PLINK v.1.9 (Chang et al., 2015) to identify and remove all SNPs in linkage disequilibrium (LD; *r* ^2^ > 0.20; *n* = 3,512 SNPs). The LD‐pruned data set included 192 individuals and 10,881 unlinked SNPs on 4,679 loci.

We then used VCFtools to calculate proportion of missing SNP calls per individual (range 1%–87%) and removed 12 individuals with >25% missing data. Because inclusion of related individuals can bias population genetic analyses (Rodríguez‐Ramilo & Wang, 2012), we estimated individual pairwise relatedness in PLINK using the identity‐by‐descent estimator PI HAT. Removal of eight individuals (6 *roselaari* E, 2 *rufa*) resolved all cases of relatedness involving half‐siblings or closer (PI HAT >0.20). The final data set included 172 individuals and 10,881 SNPs. We used VCFtools, PLINK, and PGDspider v.2.1.0.3 (Lischer & Excoffier, 2012) to convert data to different formats required for analysis.

### 2.3 Inference of population structure and diversity

To assess major axes of genetic variation and clustering among samples, we performed a principal component analysis (PCA; Patterson et al., 2006) using the R packages *gdsfmt* v.1.14.1 and *SNPRelate* v.1.12.2 (Zheng et al., 2012). We estimated ancestry proportions of each individual using the model‐based clustering procedure of ADMIXTURE v.1.3.0 (Alexander et al., 2009). We performed 10 replicate runs (with random seeds) for each putative number of ancestral populations (*K*) ranging from 1 to 8. We assessed how the cross‐validation (CV) error rate varied with increasing *K* as a first assessment of the best *K* (Alexander & Lange, 2011). However, estimating *K* is known to be a difficult issue (Novembre, 2016) and this CV criterion alone is usually unable to separate closely related populations (Alexander & Lange, 2011; Lawson et al., 2018). Therefore, following the recommendations of Pritchard et al. (2010), and Lawson et al. (2018), we here used ADMIXTURE as an exploratory tool, inspecting newly created clusters and the stability of admixture proportions with increasing *K*, and compared the results with those from the PCA and with prior biological knowledge. We used the CLUMPAK (Cluster Markov Packager Across K; Kopelman et al., 2015) web server (http://clumpak.tau.ac.il/) with default settings to summarize estimates of individual ancestry proportions to each cluster across replicate runs, and visualize the most likely ancestry proportions at each value of *K*.

Globally and for each putative population, we characterized genetic diversity by calculating per‐site nucleotide diversity (π), heterozygosity, and inbreeding coefficient (*F*_IS_) using VCFtools. To detect deviations from mutation‐drift equilibrium, we estimated the per‐locus Tajima's *D* values for each population using VCFtools. We estimated genetic differentiation among populations by calculating pairwise *F*_ST_ (Weir & Cockerham, 1984) using the diffCalc function in the R package *diveRsity* v.1.9.90 (Keenan et al., 2013), with 95% confidence intervals (CI) derived from 500 bootstraps. We calculated *p*‐values for *F*_ST_ estimates using the pairwiseTest function (1,000 permutations) in the R package *strataG* v.2.4.905 (Archer et al., 2017).

### 2.4 Population evolutionary relationships and demographic history

To visualize evolutionary relationships among populations, we first constructed an unrooted neighbour‐joining (NJ) tree based on Nei's genetic distance (Nei, 1972) using the R packages *poppr* v.2.8.5 (Kamvar et al., 2014) and *ape* v.5.3 (Paradis & Schliep, 2019), with missing data replaced by mean allele counts, and node support calculated from 1,000 bootstraps.

We used TreeMix v.1.13 (Pickrell & Pritchard, 2012) to explore the most likely population topology while accounting for possible gene flow or admixture among branches. Using genome‐wide allele frequency data, TreeMix estimates a maximum‐likelihood tree of populations with the nodes and branch lengths representing the amount genetic variance (or drift) shared among populations and within each one, respectively. Migration edges are added in a stepwise manner among populations, minimizing the genetic covariance unexplained by the tree. We performed 10 replicate TreeMix runs for each value of *m* (migration edges) from 1 to 10, and evaluated the change in log‐likelihood as *m* was increased (Δ*m*) using the R package *OptM* v.0.1.3 (Fitak, 2019) to determine an optimal *m* value ranging from two to four. For each value of *m* (2–4), we then performed 1,000 bootstrap replicates in TreeMix using blocks of 500 SNPs to derived a consensus population tree topology with node supports using the R package *BITE* v.1.2.0008 (Milanesi et al., 2017). A final run was conducted using the consensus tree and the optimized number of migration edges (*m* = 2, 3 or 4) using the full data set and the options “‐tf consensus.tree –se”, to generate the final population graph with bootstrapped node supports as described in Milanesi et al. (2017).

We evaluated the support for different possible scenarios of population divergence and admixture using the approximate Bayesian computation (ABC) (Beaumont et al., 2002) random forest (RF) statistical framework (Pudlo et al., 2016; Raynal et al., 2019). ABC‐RF can estimate posterior probabilities of historical scenarios, based on coalescent simulations of genetic data. Simulations are compared to observed data using summary statistics to identify the best‐fitting model by calculating the number of RF votes and to derive the posterior probability for the best model. The best‐fitting posterior parameter distribution values for the best model can be estimated using a RF procedure applied in a regression setting (Raynal et al., 2019). Estimated parameters include the effective size (*N*_e_) for each population, split times among populations (*t*), and the timing (*t*) and rates (*r*) of admixture events. We reduced the data set to all 4,126 SNPs genotyped in at least one individual per population. We then evaluated potential evolutionary scenarios in a stepwise manner, as follows.

In Step 1, we compared four scenarios (Figure S1, Table S2), aimed at resolving the backbone population topology, before further exploration of evolutionary scenarios. The first three scenarios (Figures S1a–c) represent three possible rootings of the unrooted topology inferred by TreeMix (see Results), including two admixture events representing the two best‐supported migration edges (i.e., admixed origins of *piersmai* and *rufa*/*islandica*). The fourth scenario (Figure S1d) represents an alternative hypothesis for the uncertain relationships among Palearctic populations: using a topology consistent with the NJ tree and TreeMix, we included admixed origins of *canutus* and *rogersi*.

In Step 2, we started with the best‐supported scenario from Step 1 (scenario *c*), and added additional complexity representing likely evolutionary scenarios (Figure S2, Table S3). First, we varied the age of the admixed origin of *piersmai* relative to other divergences (Figures S2a,b). Then, to each of these scenarios, we added admixture events representing the third (c,d) and fourth (e,f) best‐supported migration edges inferred by TreeMix (i.e. admixed origins of *islandica* and *roselaari* E). For comparison, we created three additional scenarios representing alternative hypotheses for the origin of present‐day populations (i.e. expert opinion); these included *islandica* diverging from *rufa* before (g,h) or after (i) admixture with the other Nearctic population *roselaari*, and an admixed origin of the Beringian populations *rogersi* (g,i) and/or *roselaari* W (g–i), rather than *piersmai* (see Figure S2).

The scenario parameters were considered as random variables drawn from prior distributions (Tables S2 and S3). We used DIYABC v.2.1.0 (Cornuet et al., 2014) to simulate 20,000 genetic data sets per scenario with the same properties as the observed data set (number of loci and proportion of missing data). Simulated and observed data sets were summarized using the whole set of summary statistics proposed by DIYABC for SNP markers, describing the genetic variation for each population (e.g., genetic diversity), pair of populations (e.g., *F*_ST_ and Nei's distances), or trio of populations (e.g., admixture statistics) (see the full list and details in Table S4). Linear discriminant analysis (LDA) components were also used as additional summary statistics (Estoup et al., 2012). The total number of summary statistics was 268 and 272 for step 1 and 2, respectively.

We used the RF classification procedure to compare the likelihood of the competing scenarios at each step with the R package *abcrf* v.1.8.1 (Pudlo et al., 2016). RF is a machine‐learning algorithm that uses hundreds of bootstrapped decision trees to perform classification, using the summary statistics as a set of predictor variables. Some simulations are not used in decision tree building at each bootstrap (i.e., the out‐of‐bag simulations), and are used to compute the “prior error rate,” which provides a direct method for estimating the CV error rate. At each step, we built a training set of 20,000 simulated data sets per scenario, with the same number of loci and individuals as the observed data set. We then grew a classification forest of 1,000 and 1,500 trees respectively for Step 1 and Step 2. The RF computation provides a classification vote for each scenario (i.e., the number of times a model is selected from the decision trees). We selected the scenario with the highest classification vote as the most likely scenario, and we estimated its posterior probability following the recommendation of Pudlo et al. (2016). We assessed the global performance of our chosen ABC‐RF scenario, by calculating the prior error rate based on the available out‐of‐bag simulations and we repeated the RF analysis 10 times to ensure that the results converged.

Then, posterior distribution values of all parameters for the best model identified were estimated using a regression by RF methodology (Raynal et al., 2019), with classification forests of 1,000 decision trees, and based on a training set of 100,000 simulations. We converted estimates for timing parameters from generations to years assuming a generation time of 6 years (delayed maturity with adult annual survival c. 0.80; Méndez et al., 2018) and the genome‐wide mutation rate calculated by Zhang et al. (2014) for Charadriiformes: 1.5 × 10^−9^ substitutions per site and per year. The simulation steps, computation of summary statistics, and model checking analysis were performed in DIYABC v.2.1.0. All scenario comparisons and estimations of parameter posterior distribution values were conducted with the R package *abcrf* v.1.8.1 (Pudlo et al., 2016; Raynal et al., 2019).

## 3 RESULTS

### 3.1 Summary of nextRAD SNP data set

The final data set comprised 172 unrelated individuals, including 7–31 individuals in each of the seven hypothesized populations (Table S1), genotyped at 10,881 unlinked high‐quality SNPs. On average, individuals were genotyped at 95.5% (range: 76%–99%) of SNPs and with a mean read depth of 57.3 (range: 17–137). Each SNP was genotyped in an average of 164.3 (range: 149–172, or 86.6%–100%) individuals. Globally, nucleotide diversity (π) was 0.219, and observed heterozygosity was 18.7% (Figure S3, Table S5).

### 3.2 Population structure and diversity

**FIGURE 2:**
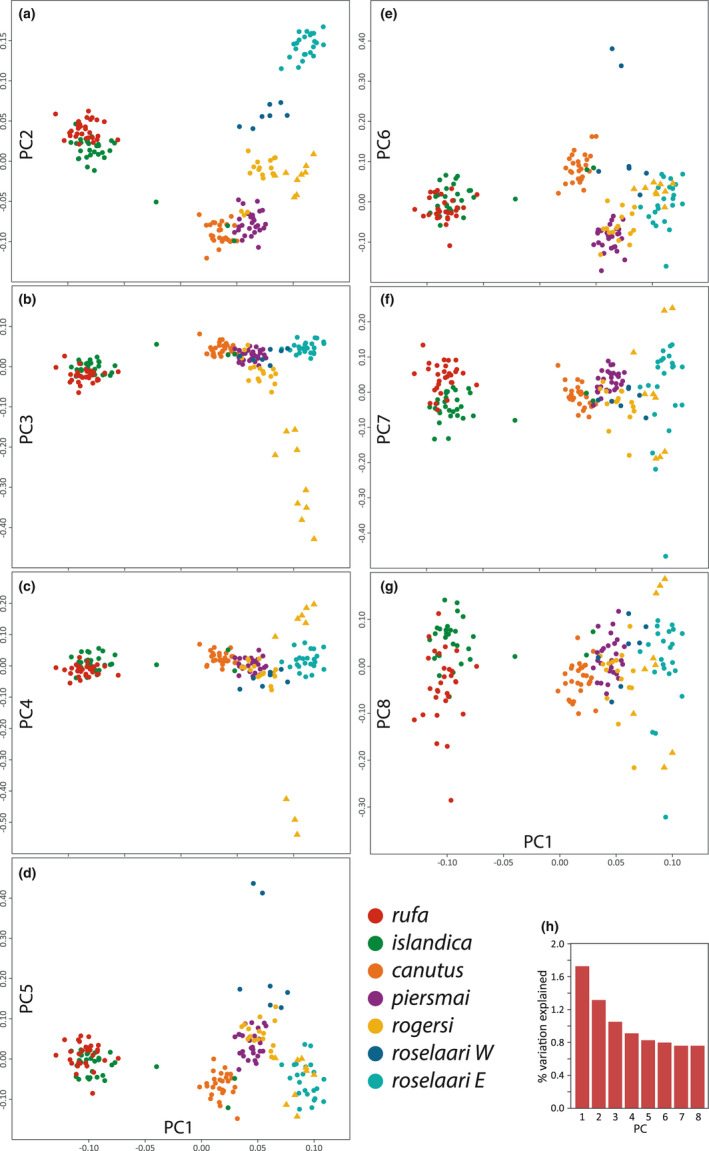
Population structure estimated by principal component analysis. The individual scores for the first principal component (PC1) are shown against the seven others (PC2–8) in panels (a) to (g). The scree plot (h) indicates the proportion of explained genetic variance by each PC, with PC1–8 explaining 8.2% of total variation. Note the nine individuals sampled in Chukotka (yellow triangles) that were distinguishable from the rest of *rogersi*. Also note two green and three yellow dots that fell among the *canutus*/*piersmai* cluster, indicating incorrect *a priori* identification of purported *islandica* and *rogersi* individuals, respectively

The first eight axes (PC1–8) of the principal component analysis explained 8.2% of the total genetic variation (Figure 2h). PC1 and PC2 distributed individuals into a triangular pattern (Figure 2a), suggesting three major genetic pools: a Canadian Arctic group of *rufa*/*islandica*, a central Palearctic group of *canutus*/*piersmai*, and a group formed by *roselaari* E from Alaska. Individuals from *rogersi* and *roselaari* W clustered in intermediate positions between the *canutus*/*piersmai* and *roselaari* E clusters, suggesting these populations may represent admixed groups between the two extreme clusters. Unexpectedly, PC3 separated nine of 13 Chukotka‐breeding individuals from the main *rogersi* cluster (Figure 2b). Higher‐order axes of variation (PC5–6) clearly distinguished *canutus* from *piersmai* (Figure 2d–e), and a subtle but noticeable separation of *rufa* and *islandica* was provided by all the PCs, especially on PC7–8 (Figure 2f–g).

**FIGURE 3:**
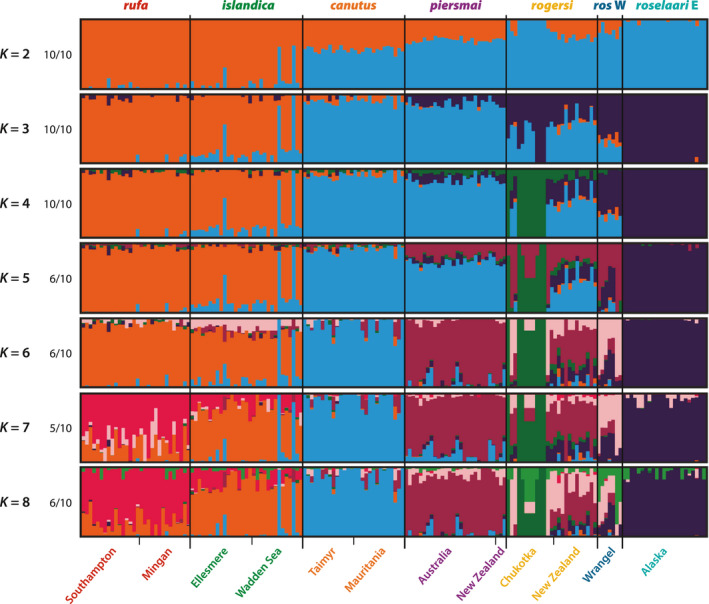
Individual genetic ancestries assigned to major clusters for *K* = 2–8 estimated using ADMIXTURE. At each value of *K*, the ancestry proportions for the 172 individuals for the dominant solution were determined by CLUMPAK summary of 10 replicate runs. Numbers on left indicate proportion of replicate runs contributing to the dominant solution (see Figure S4b for minor clusters for K = 5–8). Names below plot indicate sampling locations (see Table S1)

Similar to the three major axes of variation identified by PCA, the CV error in the genetic ancestry analysis of ADMIXTURE conservatively suggested that there could up to three ancestral genetic pools (Figure S4a). Major clusters at *K* = 2–4 had 100% support among 10 replicate runs, and greater values of *K* demonstrated further sub‐structuring consistent with the PCA analysis, geography, or *a priori* hypotheses of population structure (see major clusters for *K* = 2–8 in Figure 3; minor clusters for *K* = 5–8 are shown in Figure S4b). Consistent with the PCA, individual ancestry estimated at *K* = 3 identified three major genetic clusters: *rufa*/*islandica*, *canutus*/*piersmai*, and *roselaari* E, with *rogersi* and *roselaari* W displaying significant amounts of admixture. Interestingly, one individual sampled in the *islandica* breeding range displayed admixed genetic ancestry between *islandica* and *canutus* at *K* = 2–5, and therefore represents a possible F1 *islandica*/*canutus* hybrid (see also Figure 2). The nine Chukotka‐breeding knots identified as distinct within the *rogersi* group on the third axis of the PCA (Figure 2b) were also recognized by the ADMIXTURE analysis as a distinct genetic cluster at *K* ≥ 4; other rogersi samples from Chukotka and New Zealand contained only small proportions of this cluster. *K* = 5 identified a cluster strongly present in Palearctic Beringian populations (*piersmai*, *rogersi*, *roselaari* W), but almost absent in Alaska (*roselaari* E). Greater values of *K* (≥6) illustrated the noticeable differentiation of *canutus* and *piersmai*, and the weak but noticeable genetic structure between *rufa* and *islandica* that was also identified in the PCA.

Both PCA and ADMIXTURE indicated that five individuals were mistakenly assigned to population based on *a priori* hypotheses, without any evidence of admixture: two purported *islandica* sampled during the winter in the Wadden Sea group with *canutus*, and three purported *rogersi* from New Zealand appear to be *piersmai* (Figures 2 and 3). Although these individuals have unknown breeding areas, we judge these to reflect errors in our assignment of nonbreeding individuals, rather than cases of true dispersal and potential gene flow. As there is no evidence these individuals contributed to the gene pool where they were *a priori* identified, we reassigned these five individuals to their “correct” or “original” populations for all subsequent analyses. Also, we hereafter consider the two *rogersi* clusters separately, with *rogersi* 2 comprising the nine Chukotka individuals differentiated in PC3, and *rogersi* 1 comprising the remaining 16 *rogersi* individuals. Aside from these cases, negligible differences between sampling locations presumed to represent the same population confirmed our *a priori* assumptions about population distributions (within *rufa*, *islandica*, *canutus*, and *piersmai*; see Figure 3, Table S6).

Globally, nucleotide diversity (π = 0.219) was similar for all populations, but slightly lower in *rogersi* 2 (π = 0.184, Figure S3a, Table S5). Mean observed heterozygosity was also generally uniform among populations, but slightly higher and more variable in *rogersi* 1 and *roselaari* W (Figure S3b). We found some evidence of inbreeding (mean *F*_IS_ > 0.22) in *rogersi* 2 and *roselaari* W (Figure S3c). Values of Tajima's *D* (range of means: 0.006–0.154) were indistinguishable from zero for all populations (Figure S3d), suggesting no strong deviations from neutral expectations.

Pairwise *F*_ST_ among the eight hypothesized populations ranged 0.005–0.058, and all differed significantly from zero (*p *< .01; Table 1). The two lowest estimates involved pairs of previously recognized subspecies: *rufa* vs. *islandica* (*F*_ST_ =0.005) and *canutus* versus *piersmai* (*F*_ST_ =0.007). For all populations, the greatest pairwise differences were with *rogersi* 2 (*F*_ST_ =0.036–0.058), including within purported *rogersi* (*rogersi* 1 vs. *rogersi* 2: *F*_ST_ =0.036). By contrast, other pairwise differences with *rogersi* 1 were lower (all ≤0.021). Comparisons with *roselaari* W also produced relatively large *F*_ST_ estimates (0.020–0.058).

**TABLE 1:** Mean population pairwise *F*_ST_ and 95% confidence intervals of estimates. All comparisons are significantly different from zero (1,000 permutations; *p < .01, **p < .001)

	*rufa*	*islandica*	*canutus*	*piersmai*	*rogersi* 1	*rogersi* 2	*roselaari* W
*islandica*	0.005**						
	[0–0.012]						
*canutus*	0.020**	0.016**					
	[0.014–0.028]	[0.009–0.023]					
*piersmai*	0.021**	0.018**	0.007**				
	[0.016–0.029]	[0.012–0.026]	[0.001–0.014]				
*rogersi* 1	0.021**	0.019**	0.013**	0.009**			
	[0.009–0.043]	[0.007–0.040]	[0–0.032]	[0–0.031]			
*rogersi* 2	0.055**	0.056**	0.047**	0.045**	0.036**		
	[0.035–0.086]	[0.037–0.086]	[0.028–0.078]	[0.025–0.075]	[0.006–0.076]		
*roselaari* W	0.032**	0.031**	0.029**	0.026**	0.020**	0.058*	
	[0.005–0.071]	[0.006–0.072]	[0.004–0.066]	[0.002–0.071]	[0–0.067]	[0.016–0.114]	
*roselaari* E	0.031**	0.031**	0.027**	0.024**	0.018**	0.052**	0.024**
	[0.024–0.044]	[0.023–0.043]	[0.019–0.039]	[0.017–0.037]	[0.004–0.042]	[0.031–0.084]	[0–0.063]

### 3.3 Population evolutionary relationships and demographic history

**FIGURE 4:**
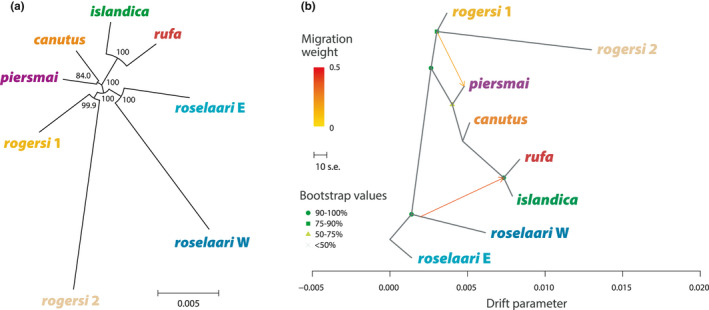
Evolutionary relationships among populations of red knots. (a) Unrooted neighbour‐joining tree, based on Nei's minimum distance with bootstrapped node confidence (%). (b) Maximum‐likelihood (ML) tree inferred by TreeMix, including two migration edges. See Figures S5 and S6 for TreeMix results with two to four migration edges

The neighbour‐joining (NJ) tree based on Nei's distance (Figure 4a) identified four closely‐related pairs of populations: *canutus*/*piersmai*, *rufa*/*islandica*, *roselaari* W/E, and *rogersi* 1/2, the last two pairs forming a Beringian group including *rogersi* and *roselaari*. These pairs clustered with high node support (>99.9%), except *canutus*/*piersmai* that had slightly lower support (84.0%). Furthermore, the long branch length between the two clusters in *rogersi* confirmed that they are distinct but closely‐related.

We further explored the population evolutionary relationships using TreeMix (Figures 4b, S5 and S6). To understand how the unexpected *rogersi* 2 cluster would affect topologies inferred by TreeMix, we ran this analysis with *rogersi* 2 either included or excluded. In both cases, change in likelihood and proportion of explained genetic variance with increasing number of migration edges (*m*) indicated strong support for at least two migrations edges (*m* = 2). Adding 3 or 4 migration edges further improved the explained variance and likelihood of the model, although the improvement was less pronounced (Figures S5 and S6); Figure 4b shows the topology inferred at *m* = 2 with *rogersi* 2 included. Across all scenarios, the most consistently supported migration edge was from the *roselaari* branch to *rufa*/*islandica*. The branching relationships among *canutus*, *piersmai*, and *rogersi* varied across scenarios, and featured low node support (≤50%), but uncertainty in this part of the topology was consistently addressed with the inference of a migration edge to *piersmai* from either *canutus* or the *rogersi* branch. The third best‐supported edge indicated migration from *canutus* to *islandica* (Figures S5 and S6). At *m* = 4, the scenarios with and without *rogersi* 2 differed in the inferred topology and migration edges, but both included a weakly‐supported migration edge from the *rogersi* branch to *roselaari* E.

**FIGURE 5:**
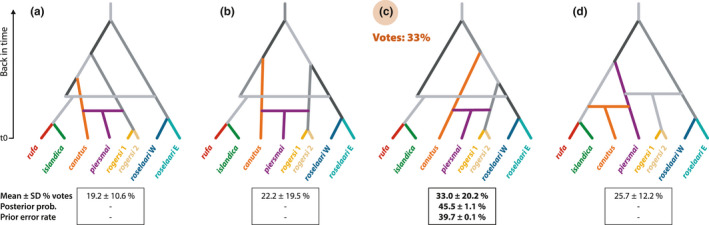
Scenarios tested in Step 1 of DIYABC analysis: (a–c) three possible rootings of the population topology inferred by TreeMix, including two admixture events (horizontal branches); (d) an alternative scenario with hypothesized admixed origin of *canutus* and *rogersi*. Extant (sampled) populations are indicated by colours; inferred historical populations are shown in grey. The best‐supported scenario was (c), as inferred by the proportion of Random Forest classification votes; for this scenario, posterior probabilities, and classification error rates are also indicated. See Figure S1 and Table S2 for further details on the model parameters

Finally, we compared the likelihood of alternative scenarios describing different population branching topologies and admixture events suggested by the above analyses using the approximate Bayesian computation – random forest approach (ABC‐RF). Step 1 of the ABC‐RF analysis (Figures 5 and S1) supported scenario *c*, representing the topology inferred by TreeMix, rooted such that the Canadian Arctic group (*rufa*/*islandica*) descended from a lineage that was the first to split from a group comprising all Palearctic and Alaskan populations (Figure 5c). This scenario received the greatest number of RF votes (33.0%), with a posterior probability of 45.5% and a prior error rate of 39.7% (Figure 5). The second best‐fitting scenario, receiving 25.7% of the RF votes, was scenario *d*.

**FIGURE 6:**
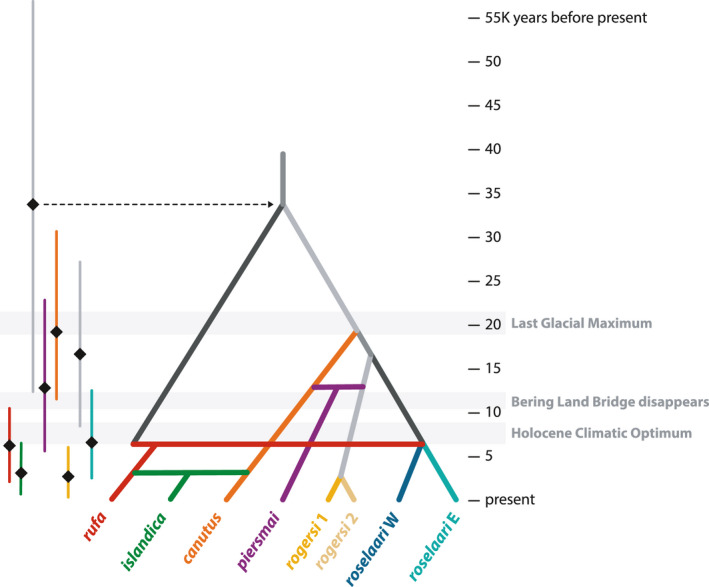
Best‐supported scenario in Step 2 of DIYABC analysis (see Figure S2), scaled to relative time‐parameter estimates (converted to years assuming a generation time of 6 years) for five divergence events (branches) and three admixture events (horizontal bars). On left, each time parameter is indicated by an estimate (black diamond) and 95% confidence interval (vertical line)

Step 2 in the ABC‐RF built upon the best‐supported scenario in Step 1 by comparing nine scenarios: six consisting of variations on scenario *c* from Step 1, each including additional admixture events as suggested by TreeMix, and three scenarios testing other plausible biogeographic hypotheses (Figure S2 and Table S3). The six closely‐related variations from scenario *c* in Step 1 (scenarios *a* to *f* in Step 2; Figure S2) received collectively 79% of the RF votes. In contrast, the alternative scenarios (*g* to *i*; Figure S2) were least supported, each receiving ≤9% of the votes. The single best‐supported scenario was *d* (Figure 6), with the greatest number of votes (17%), a posterior probability of 43.9%, and a prior error rate of 31.4% (Figure S2). This scenario included an older admixed origin of *piersmai* and an additional recent admixed origin of *islandica*; this reflects support for the third, but not the fourth, inferred migration edge from TreeMix. However, the second best‐supported scenario (*f*, with 15% of votes; Figure S2), included the fourth TreeMix migration edge, providing weak support for recent admixture between *rogersi* and *roselaari*.

Each of the 25 demographic parameters of the best‐supported scenario was estimated within the ABC‐RF framework (Table S7). Timing parameter estimates support a pre‐LGM divergence of the Canadian Arctic group from the Palearctic/Beringian group (mean estimate 33,718 ybp, 95% CI: 12,436–56,718; Figure 6, Table S7), followed by post‐glacial divergences and admixture in both major branches. In particular, we infer recent divergences within *roselaari* (6,722 ybp, CI: 2,677–12,516) and *rogersi* (2,830 ybp, CI: 534–6,078), and two instances of secondary contact between the Nearctic and Palearctic groups, via *roselaari* (6,356 ybp, CI: 2,291–10,518) and via *canutus* (3,242 ybp, CI: 870–6,528; Figure 6). Estimated effective population sizes of the eight extant populations (N1–8 in Table S7) ranged from 4,231 to 50,616 and captured some expected differences among populations. For example, the three lowest *Ne* estimates were for a highly endangered population (*rufa*) and two presumably small populations with signals of inbreeding and lower than expected heterozygosity (*rogersi* 2, *roselaari* W; Figures S3b–c). Conversely, the two admixed populations (*islandica*, *piersmai*) produced the highest *N*_e_ estimates. Estimated admixture rates were also consistent with results of ADMIXTURE and TreeMix: *islandica* derived 67% of its ancestry from *rufa* and 33% from *canutus* (r1 in Table S7), whereas *piersmai* derived 33% from *canutus* and 67% from *rogersi* (r2).

## 4 DISCUSSION

This study substantially revises our understanding of the nature, age, and origins of the current global distribution of red knots. Our historical reconstruction strongly supports the persistence of the species in two refugia at the LGM, followed by at least two instances of admixture after recent secondary contact between Nearctic and Palearctic lineages. In addition, we detected unrecognized structure within two populations currently regarded as subspecies. Below, we discuss the implications of our results for understanding: (1) the impacts of post‐LGM environmental changes, (2) present‐day population structure, and (3) the potential drivers of isolation and gene flow among flyway populations.

### 4.1 Post‐glacial phylogeography of red knots

Previous population‐genetic work in red knots (Buehler & Baker, 2005) suggested that the species was restricted to the Palearctic during the LGM, and only colonized the Canadian Arctic in the last few thousand years, after the Holocene Climatic Optimum (Buehler et al., 2006). This conclusion was partly based on extremely low differentiation at the mtDNA control region among the three Nearctic subspecies (Buehler & Baker, 2005). However, the apparent close relationship of *roselaari* to *rufa* and *islandica* was an artefact of sampling – in fact, there were likely no *roselaari* in that study, as the purported *roselaari* samples came from the southeastern USA, now considered an exclusive *rufa* wintering area (Atkinson et al., 2005; Carmona et al., 2013; Verkuil et al., 2021). Using verifiable breeding samples, we identified *roselaari* and *rufa* as the most differentiated neighbouring population pair, making a scenario of eastward colonization of the Nearctic (Buehler et al., 2006) highly unlikely. This leaves two plausible scenarios: (1) after the LGM, *canutus* spread from Europe to colonize the Nearctic breeding range of *islandica*, and then gave rise to *rufa* through a southward expansion from Ellesmere Island; or (2) the *rufa*/*islandica* clade arose *in situ* from a Nearctic refugium. The former scenario is possibly consistent with estimates of *F*_ST_ (Table 1) and Nei's distance (Figure 4a), which suggest a closer relationship of *rufa*/*islandica* with the western Palearctic (*canutus*/*piersmai*) than with the Beringian group (*rogersi*/*roselaari*). However, the explicit consideration of admixture (Figures 3 and 4b) supports the latter scenario, in which a previously diverged Nearctic branch came into recent secondary contact with both western Palearctic and Beringian groups. Our DIYABC analysis corroborated this, indicating that a Nearctic ancestor diverged from the ancestor of the Palearctic/Beringian clade c. 34,000 ybp, and then experienced admixture from both the west and east in the last few thousand years (Figure 6). For comparison, the oldest population divergence estimated by Buehler and Baker (2005) was 20,000 ybp (95% CI: 5,600–58,000), consistent with an entirely post‐LGM expansion from the Palearctic. The deeper history of divergences we describe suggests that red knots survived the LGM in both Palearctic and Nearctic refugia.

It is well established that a huge swath of the Arctic including most of Beringia and stretching westward almost to the Taimyr Peninsula in central Russia was largely ice‐free at the LGM (Ehlers & Gibbard, 2007; Pielou, 1991), and this refugium probably gave rise to all Palearctic and Beringian red knot populations. Meanwhile, the Nearctic was predominantly glaciated at the LGM, including nearly all of the present‐day breeding ranges of *islandica* and *rufa*. However, multiple lines of evidence support potential refugia in this region (Dyke, 2004; Fedorov & Stenseth, 2002; Provan & Bennett, 2008), including on Banks Island near the western end of the *rufa* range (Figure 1) and in northeast Greenland within the *islandica* range. Predictive modeling of habitats at the LGM suggested that Banks Island was most suitable for tundra‐breeding shorebirds in the Canadian Arctic, whereas ice‐free areas at higher latitudes were less‐suitable polar desert (Arcones et al., 2020). Much of the present‐day high‐Arctic breeding range of *islandica* was not ice‐free until c. 6,000 ybp (Dyke, 2004). Our DIYABC analysis inferred that *islandica* arose c. 3,200 ybp, after Nearctic red knots had established contact with *roselaari* ca. 6,400 ybp (Figure 6), perhaps through non‐breeding overlap in temperate North America. Thus, it is most likely that red knots persisted at the LGM in a refugium on or near Banks Island, and then expanded northeastward as glaciers retreated, colonizing the present‐day *islandica* range. After this, a new migration route to Europe was established, promoting contact and admixture with *canutus* in the last 3,000–4,000 years (Figure 6).

At the LGM, a southward migration from Banks Island to temperate or tropical non‐breeding areas would require a flight of >2,500 km over the Laurentide Ice Sheet, which covered much of central North America (Dyke, 2004; Ehlers & Gibbard, 2007). Present‐day migrations of all red knot subspecies involve non‐stop flights of 4,000–10,000 km (Conklin et al., 2017). In particular, *islandica* individuals can fly 4,000 km from the Canadian Arctic to western Europe, including up to 1,700 km across the Greenland Ice Sheet (Kok et al., 2020). Therefore, we find a migration from Banks Island entirely plausible. Similarly, the historical migration of the westernmost Palearctic red knots (i.e., present‐day *canutus*) likely involved flights across the ice sheets that covered northwestern Europe (Batchelor et al., 2019), which could explain their contemporary non‐stop flights between Taimyr and the Wadden Sea coast (Figure 1), both of which were largely ice‐free at the LGM (Ehlers & Gibbard, 2007).

As recognized by Buehler et al. (2006), the post‐LGM diversification of at least four Palearctic/Beringian red knot populations (Figure 6) is consistent with a scenario of increasingly isolated patches of tundra breeding habitat in the warming period prior to the Holocene Climatic Optimum (Arcones et al., 2020; Kraaijeveld & Nieboer, 2000; Stewart & Dalén, 2008; Wauchope et al., 2017). The inferred admixed origin of *piersmai* (Figure 6) suggests that a population on the New Siberian Islands (Figure 1) received subsequent immigration from two previously isolated mainland populations (*canutus* and *rogersi*). Interestingly, unlike much of the present‐day *rogersi* breeding range, the region of our Chukotka sampling site was at least partially glaciated at the LGM (Ehlers & Gibbard, 2007; Gualtieri et al., 2000), suggesting that the unexpected genetic cluster we detected (*rogersi* 2) could reflect a recent colonization of this disjunct breeding area (Figure 1). The warming period of retreating glaciers also featured rising sea levels which inundated the Bering Land Bridge, gradually isolating Alaska from Chukotka (and thus *roselaari* E from *rogersi*) by c. 12,000 ybp, and eventually Wrangel Island from mainland Russia (i.e., *roselaari* W from *rogersi*), ca. 10,000 ybp (Dyke, 2004; Manley, 2002). The population structure and uncertain tree topology we found in the eastern Palearctic attests to a recent history of climate and habitat upheavals in the region (McLaughlin et al., 2020). Expanded and more targeted genetic sampling may reveal further insights into this complicated regional history.

### 4.2 Further implications for global population structure

We confirm the close relationship between the Nearctic subspecies *rufa* and *islandica*, which were effectively indistinguishable in mtDNA (Buehler & Baker, 2005). With genome‐wide SNPs, we found weak but significant differentiation, which may reflect a recent northeastward expansion (see above), but raises the question of whether the populations are demographically independent. Currently, the extent and potential overlap of the breeding ranges of *rufa* and *islandica* are poorly described (Lathrop et al., 2018), and mark‐resight studies have recorded individuals apparently “switching” between the two flyways (Wilson et al., 2010). We sampled the two populations at the geographic extremes of the Canadian breeding range (Figure 1), and thus intermediate sampling might reveal more clinal variation by latitude across the ranges of *rufa* and *islandica*. Moreover, amplified fragment length polymorphism (AFLP) markers have indicated structure across the nonbreeding range of *rufa* (Verkuil et al., 2021), raising the possibility of longitudinal structure across the low Canadian Arctic. We note that our *rufa* samples came from easterly breeding and passage sites, and therefore do not include *rufa* that migrate through central North America (Figure 1; Newstead et al., 2013). This may also exaggerate the observed genetic differentiation between *rufa* and *roselaari* E, which are known to share nonbreeding areas in the Gulf of Mexico and perhaps western South America (Figure 1; Carmona et al., 2013; Gherardi‐Fuentes et al., 2021). Elucidating this recent, perhaps dynamic, structure requires a greater breadth of sampling across the Canadian Arctic and Greenland, which is most feasible by tracking the migrations of knots captured at nonbreeding sites.

Red knots breeding in Alaska and on Wrangel Island have been considered one subspecies, *roselaari*, due to morphological similarity and a common migration route along the west coast of North America (Carmona et al., 2013; Tomkovich, 1992). We confirmed their close relationship by descent (Figure 4), but also observed substantial genetic differentiation (Table 1). Marked individuals from both breeding areas have been detected as far south as Sonora and Baja California, Mexico (Carmona et al., 2013), suggesting that *roselaari* W and E may largely overlap during the nonbreeding season, and that population estimates and conservation efforts therefore conflate two potentially independent demographic units. Given the distinct clustering of the two populations (Figures 2 and 3), large‐scale molecular population assignment of individuals from the *roselaari* non‐breeding range may help to elucidate the population sizes and degree of year‐round spatial overlap of Wrangel Island and Alaskan knots.

The unexpected structure we detected within *rogersi* warrants further investigation. Our expectation was that nonbreeding *rogersi* samples from New Zealand and breeding samples from Meinypilgyno, in southeast Chukotka, would equally represent the population that breeds widely across northern Chukotka (see Tomkovich et al., 2013; Zöckler & O’Sullivan, 2005). However, only four of 13 Meinypilgyno individuals clustered with the New Zealand birds, while the remaining nine formed a distinct cluster (Figures and 3), and pairwise *F*_ST_ argued against a homogeneous population (Table S7). Meinypilgyno is located in a small, disjunct part of the breeding range, separated by >200 km from the closest known *rogersi* breeding areas (Figure 1; Lappo et al., 2012) and likely colonized after retreat of glaciers in the region (see above). In this light, the signals of inbreeding (Figure S3) and drift (i.e., long branch length in Figure 4) we found in *rogersi* 2 suggest a local founder effect, perhaps followed by recent immigration to the area by *rogersi* 1. Further genetic sampling and migration tracking of knots from the main *rogersi* breeding range are needed to understand this intriguing structure.

Due to the lack of sufficient breeding samples for all populations, we used nonbreeding samples when current understanding allowed confident assignment to population. These assumptions were generally confirmed (Table S7), but two exceptions provide insight about unrecognized subspecies overlap in the nonbreeding season. First, it is thought that red knots spending the winter in Europe are *islandica*, because *canutus* only passes through this region en route to nonbreeding sites in western Africa (Dick et al., 1987). However, we identified two apparent *canutus* individuals among knots sampled in the Dutch Wadden Sea in mid‐winter (14% of samples; Figures 2 and 3), implying that the wintering range of *canutus* extends into western Europe and overlaps with that of *islandica*. This has important implications for estimating populations sizes and trends based on distinct wintering areas (van Roomen et al., 2015), and provides a simpler potential pathway for gene flow, as indicated by TreeMix and the one apparent F1 hybrid (*canutus* × *islandica*) sampled at the *islandica* breeding area on Ellesmere Island (see Figure 3).

Similarly, we identified three apparent *piersmai* in our sample of purported *rogersi* from New Zealand (Figures 2 and 3). These subspecies overlap clinally during the boreal winter, such that sites in western Australia contain >80% *piersmai*, whereas sites in New Zealand are ~80% *rogersi* (Piersma et al., 2021; Tomkovich & Riegen, 2000; Verhoeven et al., 2016). Plumage differences are considered sufficient to distinguish the subspecies (Hassell et al., 2011; Tomkovich, 2001), and we correctly verified the samples of *piersmai* from Broome, Australia, based on plumage recorded during resights of these individuals when migrating through Bohai Bay, China (Rogers et al., 2010). The identification of three unrecognized *piersmai* in our New Zealand sample of purported *rogersi* (Figures 2 and 3) demonstrates that not all *piersmai* individuals attain a distinguishable plumage prior to northward migration, and can thus be mistaken for the duller‐plumaged *rogersi* subspecies at that time of year.

### 4.3 Implications for the flexibility and isolating function of migratory phenotypes

The study of population structure and differentiation in migratory taxa is particularly intriguing, due to the presumably contradictory influences of high mobility and increased phenotypic specialization (Winker, 2010). On one hand, high dispersal and the ability to cross potential geographic barriers, such as oceans and mountain ranges, should promote gene flow and weaken structure among migratory bird populations. On the other hand, selection for flyway‐specific adaptations should reduce both gene flow and successful colonization of new geographic areas. In migratory birds, fitness depends on a multi‐trait phenotype (encompassing flight, fueling, navigation, timing, molt, etc.; Åkesson et al., 2017; Piersma et al., 2005), of which some components, such as migration direction and circannual rhythms, are to some extent heritable and endogenously entrained (Berthold & Helbig, 1992; Piersma, 2011). Despite the inflexibility this may imply, novel migration behavior can evolve rapidly in new circumstances (Able & Belthoff, 1998; Berthold et al., 1992), and migratory differences may arise and persist in sympatry without precluding gene flow (Delmore et al., 2020; Pérez‐Tris et al., 2004).

In red knots, we found the weakest genetic differentiation precisely where neighboring populations display the greatest phenotypic differences: between *rufa* and *islandica*. These subspecies migrate, respectively, the longest (up to c. 15,000 km one‐way) and shortest (c. 3,000–4,000 km) distances in the species (Figure 1), differ markedly in migration direction, body size, and plumage (Buehler & Piersma, 2008; Piersma, 2011), and spend most of the year in opposite hemispheres. Conversely, we found clear structure between *roselaari* W and E, which have no obvious barriers to gene flow, as they are morphologically similar (Tomkovich, 1992) and share a flyway, directly meeting at both passage and wintering sites (Carmona et al., 2013) and migrating in essentially the same direction (Figure 1). Further understanding of the spatial organization of neutral genetic variation in red knots and other migratory birds will require quantification of the interacting contributions of history, geography, ecology, and behaviour to the formation of corridors and barriers to gene flow. This would be best accomplished in a comparative framework including multiple globally‐distributed species, and with explicit consideration of the life stage (i.e., naïve first‐time migrants vs. experienced adults) during which dispersal is most likely to occur.

## CONFLICT OF INTEREST

The authors declare no conflict of interest.

## AUTHOR CONTRIBUTIONS

J.R.C, Y.I.V, M.C.F and T.P conceived and designed the study. P.F.B, C.J.H, J.t.H, James J.A.M, and P.S.T organized and performed field sampling and maintained individual resight‐history databases. J.R.C and Y.I.V curated samples and conducted the labwork. J.R.C and M.C.F analysed and interpreted the data, with assistance from Y.I.V. J.R.C and M.C.F wrote the manuscript, with major contributions from Y.I.V and T.P. All authors provided feedback and edited the manuscript.

### OPEN RESEARCH BADGES

This article has earned an Open Data Badge for making publicly available the digitally‐shareable data necessary to reproduce the reported results. The data is available at 10.5061/dryad.j3tx95xgb.

## Supporting information

Supplementary Material

## Data Availability

Demultiplexed nextRAD short‐read data with sample metadata have been deposited in NCBI’s SRA archives under BioProject ID PRJNA799587 (accession numbers SAMN25275563–SAMN25275765). Associated files (VCF and metadata) have been deposited in DRYAD (https://doi.org/10.5061/dryad.j3tx95xgb).
