# Supplementary material for: Global flyway evolution in red knots Calidris canutus and genetic evidence for a Nearctic refugium
Source: Mol Ecol. 2022 Feb 15;31(7):2124–39. doi: 10.1111/mec.16379 (PMC9545425; doi:10.1111/mec.16379)
Supplement: Supplementary file 1 — Supplementary Material [file MEC-31-2124-s001.docx]

**SUPPLEMENTARY FIGURES & TABLES**

**Figure S1.** Scenarios tested in Step 1 of DIYABC analysis: **(a–c)** three possible rootings of the population topology inferred by TreeMix, including two admixture events (horizontal branches); **(d)** an alternative scenario with hypothesized admixed origin of *canutus* and *rogersi*. See Table S2 for all parameters and prior distributions included in the models. Extant (sampled) populations are indicated by colors; inferred historical populations are shown in gray.

**Figure S2.** Nine scenarios tested in Step 2 of DIYABC analysis. Based on the best-supported scenario in Step 1 (scenario c in Figures 5 and S1), six scenarios representing variations on the results from TreeMix were generated. Two scenarios (with two migration edges) were created to test whether the admixed origin of *piersmai* was very recent **(a)** or much older **(b)**. To each of those scenarios, we successively added the third **(c,d)** and fourth **(e,f)** migration edges inferred by TreeMix (see Figures S5 and S6). For comparison, we generated three additional scenarios representing alternative hypotheses: (1) that *islandica* diverged from *rufa* either before **(g,h)** or after **(i)** admixture with *roselaari*; and (2) that *roselaari* W **(h)** *and rogersi* **(g,i)**, rather than *piersmai*, arose from admixture within Beringia. Extant (sampled) populations are indicated by colors; inferred historical populations are shown in gray. See Table S3 for all parameters and prior distributions included in the models. The best-supported scenario was **(d)**, as inferred by the proportion of Random Forest classification votes. The posterior probability and prior error rate for this scenario is also provided. See Table S5 for all posterior distributions estimated from this scenario.

**Figure S3.** Global and per-population genetic diversity. **(a)** Nucleotide diversity (π). Sample sizes are shown across top of panel. **(b)** Observed heterozygosity, with expected heterozygosity indicated by blue bars. **(c)** Inbreeding coefficient (*F*_IS_). **(d)** Per-locus Tajima’s *D*. Values for *rogersi* and *roselaari* are each separated into two inferred genetic clusters. Boxplots show median and 25^th^ and 75^th^ percentiles, with extreme values indicated by whiskers. Mean values indicated by black diamonds.

**Figure S4.** **(a)** Cross-validation error values of the ADMIXTURE analysis for number of clusters (*K*) ranging from 1 to 8, with 10 replicate runs per *K* value. **(b)** Minor clusters for *K* = 5–8 determined by CLUMPAK summary of 10 replicate runs. Numbers on left indicate proportion of replicate runs represented by the minor cluster. Names below plot indicate sampling locations (see Table S1).

**Figure S5.** TreeMix analysis with *rogersi* 2 included. **(a)** Number of migration edges evaluated in *optM* (Evanno method; 10 replicate runs of 1–10 migration edges). Based on greatest increase in log likelihood (lower panel), inclusion of two migration edges is best-supported. Inferred population topologies and admixture events including **(b)** two, **(c)** three, or **(d)** four migration edges. For each scenario, the matrix on the right indicates scaled pairwise-population residual fit to the maximum likelihood tree.

**Figure S6.** TreeMix analysis with *rogersi* 2 excluded. **(a)** Number of migration edges evaluated in *optM* (Evanno method; 10 replicate runs of 1–10 migration edges). Based on greatest increase in log likelihood (lower panel), inclusion of two migration edges is best-supported. Inferred population topologies and admixture events including **(b)** two, **(c)** three, or **(d)** four migration edges. For each scenario, the matrix on the right indicates scaled pairwise-population residual fit to the maximum likelihood tree.

**Table S1.** Sampling details for global red knot populations. *n* Initial = samples sequenced for *de novo* SNP discovery. *n* Final = samples included in analyses after data filtering.

| **Population** | **Sampling location** | **Country** | **Month** | **Year(s)** | | **Type**^1^ | | | **Storage**^2^ | | | **Source**^3^ | | | ***n* Initial** | | | ***n* Final** |
| --- | --- | --- | --- | --- | --- | --- | --- | --- | --- | --- | --- | --- | --- | --- | --- | --- | --- | --- |
| *canutus* | Taimyr Peninsula | Russia | Jul | 1994–1995 | | B | | | BE | | | 1 | | | 16 | | | 14 |
|  | Banc d’Arguin | Mauritania | Nov–Dec, Apr | 2008–2009 | | R | | | BE | | | 2 | | | 15 | | | 14 |
|  |  |  |  |  | |  | | |  | | |  | | |  | | |  |
| *piersmai* | Roebuck Bay, WA | Australia | Jun–Nov | 2007–2008 | | PL | | | BE | | | 3 | | | 30 | | | 25 |
|  | Foxton Beach, N Isl. | New Zealand | Nov | 2013 | | PL | | | BQ | | | 4 | | | 2 | | | 2 |
|  | Foxton Beach, N Isl. | New Zealand | Nov | 2013 | | G | | | BQ | | | 4 | | | 1 | | | 1 |
|  |  |  |  |  | |  | | |  | | |  | | |  | | |  |
| *rogersi* | SE Chukotka | Russia | Jul | 2009–2010 | | B | | | OE | | | 5 | | | 2 | | | 2 |
|  | SE Chukotka | Russia | May–Jul | 2009–2014 | | B | | | BE | | | 5 | | | 17 | | | 11 |
|  | Foxton Beach, N Isl. | New Zealand | Nov, Jan | 2013–2014 | | PL | | | BQ | | | 4 | | | 7 | | | 7 |
|  | Foxton Beach, N Isl. | New Zealand | Nov, Jan | 2013–2014 | | G | | | BQ | | | 4 | | | 6 | | | 5 |
|  |  |  |  |  | |  | | |  | | |  | | |  | | |  |
| *roselaari* W | Wrangel Island | Russia | Jul–Aug | 2007 | | B | | | BE | | | 5 | | | 11 | | | 7 |
|  | Wrangel Island | Russia | Jul | 2007 | | B | | | FE | | | 5 | | | 1 | | | 0 |
|  |  |  |  |  | |  | | |  | | |  | | |  | | |  |
| *roselaari* E | Seward Pen., AK | USA | May–Jul | 2012–2014 | | B | | | BF | | | 6 | | | 30 | | | 23 |
|  |  |  |  |  | |  | | |  | | |  | | |  | | |  |
| *rufa* | Southampton Isl., NU | Canada | Jun–Jul | 1999–2002 | | B | | | BE | | | 1 | | | 19 | | | 16 |
|  | Mingan Arch., QC | Canada | Jul | 2009 | | P | | | BE | | | 1 | | | 14 | | | 14 |
|  |  |  |  |  | |  | | |  | | |  | | |  | | |  |
| *islandica* | Ellesmere Isl., NU | Canada | Jun-Jul | 1986–1987 | | B | | | OE | | | 1 | | | 7 | | | 7 |
|  | Ellesmere Isl., NU | Canada | Jun | 1990 | | B | | | BE | | | 1 | | | 11 | | | 10 |
|  | Wadden Sea | Netherlands | May-Mar | 2001–2012 | | R | | | BE | | | 2 | | | 14 | | | 14 |
|  |  |  |  |  | |  | | |  | | |  | | | **203** | | | **172** |
|  |  |  |  |  |  | |  | | |  | | |  | | |  | | |
| ^1^B | Captured or collected at known breeding area | | |  |  | |  | | |  | | |  | | |  | | |
| G | Captured at non-breeding site and tracked to breeding area by geolocator | | | | | | |  | | |  | | |  | | |  | |
| R | Captured at non-breeding site and assigned to breeding area based on individual resight history and known population movements | | | | | | | | | | | | | | | | | |
| PL | Captured at non-breeding site and assigned to breeding area based on individual plumage and known population movements | | | | | | | | | | | | | | | | | |
| P | Captured at non-breeding site and assigned to breeding area based on known population movements | | | | | | | | | | | | | | | | | |
| ^2^BE | Blood from live capture stored in 95% ethanol | | | | | | | | | | | | | | | | | |
| BQ | Blood from live capture stored in Queen's lysis buffer | | | | | | | | | | | | | | | | | |
| BF | Blood from live capture stored on filter paper | | | | | | | | | | | | | | | | | |
| FE | Feather from live capture stored in 95% ethanol | | | | | | | | | | | | | | | | | |
| OE | Organ tissue from collected specimen stored in 95% ethanol | | | | | | | | | | | | | | | | | |
| ^3^1 | Royal Ontario Museum, University of Toronto, Canada | | | | | | | | | | | | | | | | | |
| 2 | NIOZ Royal Netherlands Institute for Sea Research, The Netherlands | | | | | | | | | | | | | | | | | |
| 3 | Shorebird LifeLines, Groningen Institute for Evolutionary Life Sciences, University of Groningen, The Netherlands | | | | | | | | | | | | | | | | | |
| 4 | Ecology Group, Massey University, Palmerston North, New Zealand | | | | | | | | | | | | | | | | | |
| 5 | Zoological Museum, Moscow Lomonosov State University, Russia | | | | | | | | | | | | | | | | | |
| 6 | Migratory Bird Management, United States Fish & Wildlife Service, USA | | | | | | | | | | | | | | | | | |

**Table S2.** Prior distribution for each parameter of the models used in Step 1 of the ABC-RF analyses. N = effective population size, A = admixture rate, T = time (in generations) of population split or admixture. See Figure S1 for parameter specifics.

| **Parameters** | **Type** | **Prior** |
| --- | --- | --- |
| N1 | N | UN ~ [10 - 10,000] |
| N2 | N | UN ~ [10 - 100,000] |
| N3 | N | UN ~ [10 - 100,000] |
| N4 | N | UN ~ [10 - 100,000] |
| N5 | N | UN ~ [10 - 100,000] |
| N6 | N | UN ~ [10 - 10,000] |
| N7 | N | UN ~ [10 - 10,000] |
| N8 | N | UN ~ [10 - 100,000] |
| N9 | N | UN ~ [10 - 100,000] |
| N10 | N | UN ~ [10 - 100,000] |
| N11 | N | UN ~ [10 - 100,000] |
| N12 | N | UN ~ [10 - 100,000] |
| N13 | N | UN ~ [10 - 100,000] |
| N14 | N | UN ~ [10 - 100,000] |
| N15 | N | UN ~ [10 - 100,000] |
| r1 | A | UN ~ [0.001 - 0.999] |
| r2 | A | UN ~ [0.001 - 0.999] |
| t1 | T | UN ~ [10 - 4000] |
| t2 | T | UN ~ [10 - 4000] |
| t3 | T | UN ~ [10 - 5000] |
| t4 | T | UN ~ [10 - 6000] |
| t5 | T | UN ~ [10 - 7000] |
| t6 | T | UN ~ [10 - 8000] |
| t7 | T | UN ~ [100 - 10,000] |
| t8 | T | UN ~ [100 - 10,000] |
| Conditions: t2>t1; t3>t2; t4>t3; t5>t4; t6>t5;t7>t6; t8>t7 | | |

**Table S3.** Prior distribution for each parameter of the models used in Step 2 of the ABC-RF analyses. N = effective population size, A = admixture rate, T = time (in generations) of population split or admixture. See Figure S1 for parameter specifics.

| **Parameter** | **Type** | **Prior** |
| --- | --- | --- |
| N1 | N | UN~[10 - 10000] |
| N2 | N | UN~[10 - 100000] |
| N3 | N | UN~[10 - 100000] |
| N4 | N | UN~[10 - 100000] |
| N5 | N | UN~[10 - 100000] |
| N6 | N | UN~[10 - 10000] |
| N7 | N | UN~[10 - 10000] |
| N8 | N | UN~[10 - 100000] |
| N9 | N | UN~[10 - 100000] |
| N10 | N | UN~[10 - 100000] |
| N11 | N | UN~[10 - 100000] |
| N12 | N | UN~[10 - 100000] |
| N13 | N | UN~[10 - 100000] |
| N14 | N | UN~[10 - 100000] |
| N15 | N | UN~[10 - 100000] |
| r1 | A | UN~[0.001 - 0.999] |
| r2 | A | UN~[0.001 - 0.999] |
| r3 | A | UN~[0.001 - 0.999] |
| r4 | A | UN~[0.001 - 0.999] |
| t1 | T | UN~[10 - 3000] |
| t2 | T | UN~[10 - 4000] |
| t3 | T | UN~[10 - 5000] |
| t4 | T | UN~[10 - 6000] |
| t5 | T | UN~[10 - 7000] |
| t6 | T | UN~[100 - 8000] |
| t7 | T | UN~[100 - 10000] |
| t8 | T | UN~[100 - 10000] |
| t9 | T | UN~[100 - 10000] |
| Conditions: t2>t1; t3>t2; t4>t3; t5>t4; t6>t5; t7>t6; t8>t7; t9>t8 | | |

**Table S4.** Vector of summary statistics describing the SNP genetic variation in DIYABC v.2.1.0 used in the ABC-RF analyses.

| **Abbreviation** | **Description** |
| --- | --- |
| *Single sample statistics for each sampled population* | |
| HP0 | Proportion of loci with zero gene diversity |
| HM1 | Mean gene diversity across polymorphic loci (Nei, 1987) |
| HV1 | Variance of gene diversity across polymorphic loci |
| HMO | Mean gene diversity across all loci |
| *Two sample statistics for each pairwise sample combination* | |
| FP0 | Proportion of loci with zero *F*_ST_ distance (Weir & Cockerham, 1984) |
| FM1 | Mean across loci of non-zero *F*_ST_ distances |
| FV1 | Variance across loci of non-zero *F*_ST_ distances |
| FMO | Mean across loci of *F*_ST_ distances |
| NP0 | Proportion of loci with zero Nei’s distance (Nei, 1972) |
| NM1 | Mean across loci of non-zero Nei’s distances |
| NV1 | Variance across loci of non-zero Nei’s distances |
| NMO | Mean across loci of Nei’s distances |
| *Admixture statistics (Choisy et al., 2004) for each combination of parental and admixed populations* | |
| AP0 | Proportion of loci with zero admixture estimates |
| AM1 | Mean across loci of non-zero admixture estimate |
| AV1 | Variance across loci of non-zero admixture estimated |
| AMO | Mean across all locus admixture estimates |

**Table S5.** Descriptive statistics (mean ± SD) per population, after re-assigning five individuals to correct population (see *Results*). Depth coverage = mean coverage per SNP; π = nucleotide diversity; Het = observed heterozygosity; *F*_IS_ = inbreeding coefficient.

| **Population** | ***n*** | **Depth coverage** | **π** | **Het** | ***F*_IS_** | **Tajima’s *D*** |
| --- | --- | --- | --- | --- | --- | --- |
| All | 172 | 57.33 ± 26.62 | 0.219 ± 0.140 | 0.187 ± 0.028 | 0.145 ± 0.128 | 0.537 ± 0.776 |
| *rufa* | 30 | 70.32 ± 28.54 | 0.217 ± 0.155 | 0.199 ± 0.025 | 0.141 ± 0.109 | 0.154 ± 0.816 |
| *islandica* | 29 | 50.14 ± 15.70 | 0.220 ± 0.154 | 0.210 ± 0.015 | 0.096 ± 0.065 | 0.150 ± 0.815 |
| *canutus* | 30 | 56.30 ± 26.11 | 0.215 ± 0.154 | 0.203 ± 0.018 | 0.109 ± 0.079 | 0.126 ± 0.826 |
| *piersmai* | 31 | 42.94 ± 15.01 | 0.217 ± 0.152 | 0.213 ± 0.012 | 0.066 ± 0.053 | 0.137 ± 0.801 |
| *rogersi* all | 22 | 67.57 ± 36.49 | 0.207 ± 0.160 | 0.183 ± 0.049 | 0.218 ± 0.210 | 0.057 ± 0.829 |
| *rogersi* 1 | 13 | 61.34 ± 40.58 | 0.214 ± 0.167 | 0.223 ± 0.042 | 0.140 ± 0.162 | 0.006 ± 0.845 |
| *rogersi* 2 | 9 | 83.68 ± 21.78 | 0.184 ± 0.189 | 0.216 ± 0.057 | 0.293 ± 0.189 | 0.130 ± 0.896 |
| *roselaari* W | 7 | 71.24 ± 23.05 | 0.206 ± 0.192 | 0.248 ± 0.079 | 0.225 ± 0.243 | 0.063 ± 0.880 |
| *roselaari* E | 23 | 56.22 ± 27.23 | 0.213 ± 0.163 | 0.207 ± 0.029 | 0.147 ± 0.120 | 0.147 ± 0.840 |

**Table S6.** Pairwise genetic differentiation expressed as *F*_ST_ among samples from different sites but presumed to belong to the same population. For four populations, negligible and non-significant differentiation justifies the pooling of different sampling sites within populations. Within purported *rogersi*, there is significant differentiation (*) between breeding samples from Chukotka and non-breeding samples from New Zealand; this largely reflects the unexpected genetic cluster found within Chukotka samples (*rogersi* 2).

| **Population** | **site1** | **site2** | **estimate** | **CI lower** | **CI upper** | **P** |
| --- | --- | --- | --- | --- | --- | --- |
| *rufa* | Southampton | Mingan | 0.000 | 0.000 | 0.024 | 0.94 |
| *piersmai* | Broome | NewZealand | 0.000 | 0.000 | 0.037 | 0.80 |
| *islandica* | Ellesmere | Wadden | 0.001 | 0.000 | 0.023 | 0.23 |
| *canutus* | Taimyr | Mauritania | 0.001 | 0.000 | 0.026 | 0.16 |
| *rogersi* | Chukotka | NewZealand | 0.017* | 0.000 | 0.055 | 0.002 |

**Table S7.** Posterior distribution for each parameter of the best-fitted model (scenario d) in the Step 2 analysis of the ABC-RF (Figure S2), based on a training reference table of 100K simulations and 1,000 RF decision trees. See Figure S1 and Table S3 for parameter specifics.

| **Param** | **Mean** | **Median** | **Quantiles 0.05** | **Quantiles 0.95** |
| --- | --- | --- | --- | --- |
| N1 | 4231.24 | 3876.00 | 1376.00 | 8114.00 |
| N2 | 44088.41 | 41128.40 | 3797.27 | 95521.33 |
| N3 | 35151.10 | 30873.00 | 11977.92 | 76973.96 |
| N4 | 50616.31 | 46410.43 | 15956.00 | 93675.27 |
| N5 | 31259.66 | 20508.00 | 1009.31 | 88544.00 |
| N6 | 4411.83 | 4122.00 | 783.00 | 9205.00 |
| N7 | 4464.81 | 4178.00 | 1390.00 | 8686.00 |
| N8 | 29676.61 | 18401.00 | 2321.50 | 89020.00 |
| N9 | 51051.10 | 52393.84 | 6942.00 | 94752.90 |
| N10 | 58805.83 | 59127.60 | 19947.93 | 95323.88 |
| N11 | 55272.08 | 52944.70 | 17566.60 | 96721.00 |
| N12 | 51268.74 | 50278.00 | 10029.02 | 95227.04 |
| N13 | 43804.87 | 39038.00 | 7969.02 | 92455.80 |
| N14 | 85073.54 | 86517.60 | 64284.23 | 98453.35 |
| r1 | 0.67 | 0.68 | 0.41 | 0.93 |
| r2 | 0.33 | 0.27 | 0.02 | 0.83 |
| r3 | 0.58 | 0.63 | 0.09 | 0.96 |
| t1 | 471.73 | 424.00 | 89.00 | 1013.00 |
| t2 | 540.37 | 488.00 | 145.00 | 1088.00 |
| t3 | 1120.41 | 1042.00 | 446.13 | 2086.00 |
| t4 | 1059.33 | 1039.00 | 381.81 | 1753.00 |
| t5 | 2154.29 | 1970.00 | 955.07 | 3819.00 |
| t6 | 2784.78 | 2586.96 | 1426.95 | 4525.00 |
| t7 | 3209.53 | 3029.00 | 1926.65 | 5101.00 |
| t8 | 5619.71 | 5226.00 | 2072.64 | 9453.00 |
| **Time converted from generations to years assuming 6yr per generation** | | | | |
| t1yr | 2830.38 | 2544.00 | 534.00 | 6078.00 |
| t2yr | 3242.22 | 2928.00 | 870.00 | 6528.00 |
| t3yr | 6722.46 | 6252.00 | 2676.78 | 12516.00 |
| t4yr | 6355.98 | 6234.00 | 2290.86 | 10518.00 |
| t5yr | 12925.74 | 11820.00 | 5730.42 | 22914.00 |
| t6yr | 16708.68 | 15521.76 | 8561.70 | 27150.00 |
| t7yr | 19257.18 | 18174.00 | 11559.90 | 30606.00 |
| t8yr | 33718.26 | 31356.00 | 12435.84 | 56718.00 |
